# Evaluation of the Removal and Effects of Cylindrospermopsin on Ripened Slow Sand Filters

**DOI:** 10.3390/toxins15090543

**Published:** 2023-09-02

**Authors:** Daniel Valencia-Cárdenas, Thatiane Souza Tavares, Rafaella Silveira, Cristina Celia Silveira Brandão, Raquel Moraes Soares, Yovanka Pérez Ginoris

**Affiliations:** Department of Civil and Environmental Engineering, Faculty of Technology, University of Brasilia, Brasilia 70910-900, Brazil; thatianest@gmail.com (T.S.T.); rsilveira.bio@gmail.com (R.S.); cbrandao@unb.br (C.C.S.B.); kelsoares@gmail.com (R.M.S.)

**Keywords:** cylindrospermopsin, slow sand filtration, *schmutzdecke*, water treatment, microbiota

## Abstract

The occurrence of toxic blooms of cyanobacteria has been a matter of public health interest due to the cyanotoxins produced by these microorganisms. Cylindrospermopsin (CYN) is a cyanotoxin of particular concern due to its toxic effects on humans. This study investigated the removal and effects of CYN in ripened slow sand filters (SSFs) treating water from Paranoá Lake, Brasilia, Brazil. Four pilot-scale SSFs were ripened and operated for 74 days. Two contamination peaks with CYN were applied along the filtration run. The improvement of any of the evaluated water quality parameters was not affected by the presence of CYN in the raw water. The SSFs efficiently removed CYN, presenting concentrations lower than 0.8 µg/L in the filtered water. The microbiota of the SSFs were dominated by protozoa of the genus *Euglypha* and amoebas of the genera *Arcella*, *Centropyxis*, and *Amoeba*, together with some groups of rotifers. These microorganisms played a crucial role in removing total coliforms and *E. coli*. In addition, CYN was not identified as a determining factor in the microbiota composition.

## 1. Introduction

The occurrence of toxic cyanobacterial blooms has increased worldwide due to climate change and the eutrophication of water bodies [1,2,3]. These blooms pose a significant risk to plants, invertebrates, animals, and humans [4,5,6]. In particular, when these blooms occur in water bodies used as water supply sources, the risk to human health becomes even more concerning.

Several reports have documented human intoxication caused by the consumption of water contaminated with cyanotoxins producing cyanobacteria in different regions of the planet. Two of the best-known cases include the outbreak of hepatoenteritis in Palm Island, Australia, in 1979, caused by cylindrospermopsin (CYN) [7], and the death of approximately 60 patients from a hemodialysis clinic in Caruaru, Brazil, in 1996, due to the presence of microcystin (MC) [8].

The most commonly occurring cyanotoxins worldwide are MC and CYN [9]. MC has been widely studied, and most of the research is related to its removal from water. On the other hand, CYN is a cyanotoxin produced by a few species of cyanobacteria. *Raphidiopsis raciborskii* is the most well-known. Another 14 cyanobacterial species have been reported as potential CYN producers: *Anabaena lapponica* [10]; *Anabaena planctonica* [11]; *Aphanizomenon flos-aquae* [12]; *Aphanizomenon klebahnii* [13]; *Aphanizomenon gracile* [14]; *Chrysosporum bergii* [15]; *Chrysosporum ovalisporum* [16,17]; *Dolichospermum mendotae* [18]; *Lyngbya wollei* [19]; *Oscillatoria* sp. [20]; *Raphidiopsis curvata* [21]; *Raphidiopsis mediterranea* [22]; *Sphaerospermopsis aphanizomenoides* [23,24]; and *Umezakia natans* [25]. Its occurrence has been of increasing concern as it can pose a risk to human and animal health through systemic damage and, especially, damage to the liver [5,26].

Given the risk posed by the presence of CYN in water supply sources, the use of appropriate treatment techniques for its removal is essential. Several water treatment techniques are available, including chemical oxidation [27] and adsorption onto activated carbon [28,29]. In the context of appropriate technologies for decentralized water treatment, slow sand filtration effectively removes cyanotoxins from water [30,31], which is particularly important in areas with frequent cyanobacterial blooms. MC is the most studied cyanotoxin in the context of this treatment technology, considering it is the most common and persistent toxin in water bodies, and its presence in drinking water can represent a risk to human health.

Studies have demonstrated MC removals higher than 95% in full-scale sand slow filters (SSFs), resulting in less than 1 µg/L concentrations in the filtered water [31]. In addition, slow sand filtration systems, with continuous or intermittent flow, are equally effective, achieving concentrations of less than 1 µg/L in the filtered water [32]. MC removal by this technology has been attributed mainly to the biodegradation mechanism [32,33].

The development of *schmutzdecke*, a biological layer essential for the performance of SSFs, is crucial in the filtration process. As the *schmutzdecke* develops during filter ripening, the efficiency of the filters increases [34].

The biologically active layer of SSFs is an ecosystem [35]. Through trophic interactions, most of the removal processes take place [36,37]. Microalgae and cyanobacteria, given their capacity for photosynthesis, absorb CO_2_, nitrates, and phosphates, in addition to producing oxygen used by other organisms to carry out their metabolic processes and decompose part of the organic matter. Bacteria oxidize and mineralize part of the organic matter, using the dissolved oxygen produced in the primary metabolism and serving as a food source for higher organisms. Protozoa and invertebrates generally feed on detritus, suspended particles in the water, bacteria, and other lower microorganisms [38]. Thus, predation by protists and invertebrates is pointed out as the primary mechanism for the removal of bacteria belonging to the coliform group, such as *E. coli* [34], and pathogens, such as *Cryptosporidium* oocysts [39,40], making it relevant to the microbiological quality of filtered water.

However, cyanotoxins, such as MC, can delay microbiological development and the growth of certain groups of microorganisms, altering the microbial communities present [41,42]. The consequence is an increase in the ripening period of SSFs. In contrast, CYN does not exhibit bactericidal effects at concentrations commonly found in aquatic environments [43]. Although the information on the impacts of CYN on the microbiota present in *schmutzdecke* is poor, the study reported by Rasmussen et al. [43] showed that this cyanotoxin could be toxic to some protozoa at concentrations between 5 and 50 µg/L. Regarding the operating time of SSFs, it is known that it is an essential factor in their efficiency. Haig et al. [44] noted that the longer operating time of the filters resulted in better removal of coliform group bacteria and higher overall efficiency.

Despite the advances in cyanotoxin removal studies using slow sand filtration, these findings indicate the need to extensively investigate SSF performance and the effects of CYN on protists and metazoans, which are directly related to the removal of microorganisms of sanitary interest, such as coliform bacteria. This study investigated the potential of ripened SSFs to remove CYN and the effects of this cyanotoxin on the performance of slow sand filtration and the benthic and planktonic communities, which are primarily responsible for improving the microbiological parameters of water quality.

## 2. Results and Discussion

### 2.1. Preliminary Tests

**Figure 1:**
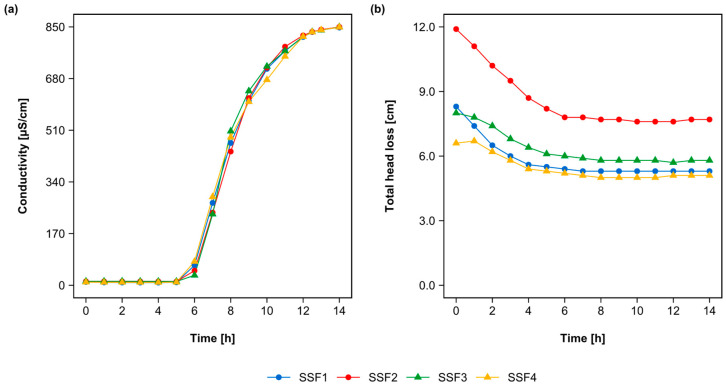
Results of the tracer and hydraulic behavior tests: (**a**) Conductivity of the tracer NaCl in the effluent of each SSF measured every 60 min over 14 h, with increased frequency (every 30 min) in the last two hours. Test carried out with initial conductivity in the effluent of 850 µS/cm. Test carried out to determine the experimental residence time of the water in each slow sand filter. (**b**) Total head loss in each SSF, monitored during the tracer test every 60 min. Test carried out to detect hydraulic differences between the SSFs.

The results of the tracer test and the hydraulic behavior test of the four SSFs are presented in Figure 1. Figure 1a shows that the water experimental residence time in the filtration system was 14 h for all the SSFs. The Kruskal–Wallis test revealed no significant differences in the residence time between the four SSFs, obtaining a *p*-value of 0.767.

Figure 1b shows the total head loss over the 14 h of the tracer test on the four SSFs. The head loss stabilized at approximately 6 h after the start of the test. As expected, all SSFs showed the same stabilization trend over time. However, despite the similar stabilization trend, SSF2 showed an average total head loss 46.6% higher than the average of SSF1, SSF3, and SSF4. This difference was confirmed by the Kruskal–Wallis test, revealing a *p*-value of 2.92 × 10^−8^. Despite this difference, the removal efficiency of the different water quality parameters and head loss did not show distinctions between the SSFs during the experiments of this study.

Furthermore, Dunn’s multiple comparisons tests indicated that the most representative differences were between SSF2 and the filters SSF1 and SSF4, with adjusted *p*-values of 5.60 × 10^−5^ and 3.01 × 10^−8^, respectively. On the other hand, the differences between SSF2 and SSF3, as well as between SSF3 and SSF4, were considered in the margin of non-significance, with adjusted *p*-values of 0.0375 and 0.0112, respectively. These differences in the total head loss between the filters may have been influenced by different factors, such as the compaction of the filter media during the installation of the SSFs or the presence of impurities in the sand used as filter media. Such factors may have directly impacted the SSF’s media porosity and, consequently, the head loss over time in the hydraulic tests.

**Figure 2:**
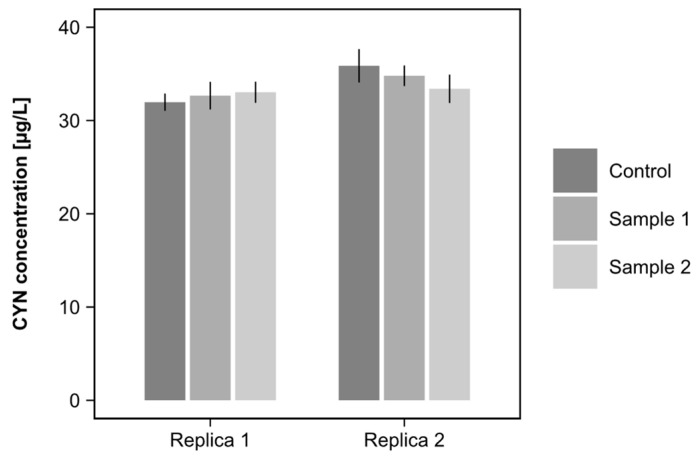
CYN concentration measured in the sand suspensions of two samples and a control used in the adsorption test on the filter media sand. Test carried out in duplicate.

The results of the evaluation of CYN adsorption on the sand used as filter media are presented in Figure 2. A similarity in the CYN concentration was observed within each replica’s samples. In replica 1, the average concentration of CYN was 32.6 µg/L. In replica 2, the average concentration was 34.7 µg/L.

For replica 1, the difference between the concentration in the samples did not exceed 2% compared to the replica mean. More specifically, the differences of each sample, relative to the mean, were −1.84%, 0.31%, and 1.23% for the control, sample 1, and sample 2, respectively. In replica 2, the differences were slightly more prominent compared to replica 1. The control, sample 1, and sample 2 showed differences compared to the mean of 3.46%, 0.29%, and −3.75%, respectively.

The results obtained were subjected to a two-way analysis of variance (two-way ANOVA), and the results are presented in Table 1. The analysis revealed that variation in the data was mainly due to the test replicas, accounting for 20.96% of the variation. However, this variation was not significant (*p*-value = 0.072). In addition, the different samples (control, sample 1, and sample 2) contributed only 1.62% of the variation in the data without statistical significance (*p*-value = 0.865). Therefore, the results indicated that the sand used as filter media did not adsorb CYN, suggesting that adsorption was not a predominant removal mechanism for CYN in this study.

### 2.2. Monitoring of Operational and Water Quality Parameters

Monitoring the slow sand filtration system operation included three periods: ripening and two contamination peaks with CYN (contamination peak 1 and contamination peak 2). Filters SSF1 and SSF2 served as control and received water from Paranoá Lake during the filtration run. On the other hand, filters SSF3 and SSF4 received lake water spiked with CYN only during the contamination peaks. This group of SSFs was already used in previous experiments, and before the beginning of this study, they were submitted to the scraping and resanding process.

The ripening lasted 42 days, while each contamination peak lasted 5 days. The first peak occurred between the 43rd and 47th day of operation and the second between the 70th and 74th. The descriptive statistics of the raw and filtered water quality parameters are presented in Table A1, Table A2 and Table A3.

In this study, the ripening period was twice as long as that reported in previous studies on Paranoá Lake water treatment by slow sand filtration [45,46,47]. This behavior is probably due to insufficient incident light on the SSFs. Such insufficiency likely inhibited the primary metabolism of phytoplankton (photosynthesis), causing dissolved oxygen lacking in the raw water column above the top of the filter media [48] and, consequently, impairing groups of aerobic microorganisms that are key to the SSFs ripening [37].

**Figure 3:**
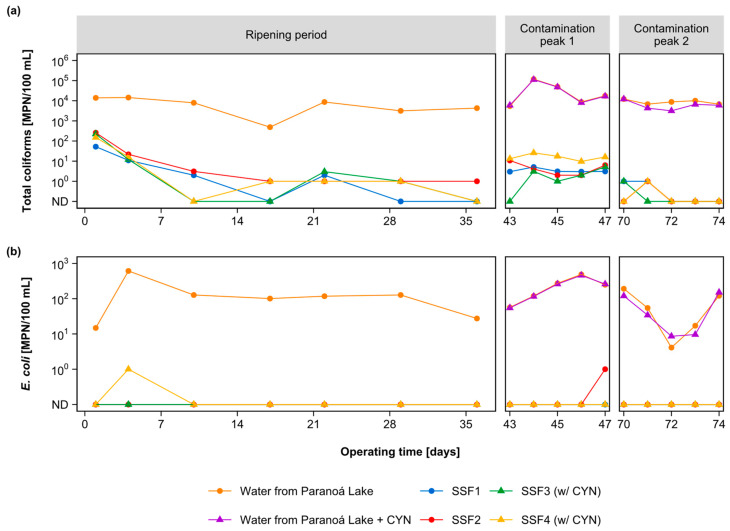
(**a**) Total coliform and (**b**) *E. coli* counts made by the Colilert test in the effluent and effluent of each SSF during the ripening period and contamination peaks 1 and 2.

Total coliform gradually reduced in all SSF effluents until the end of the ripening period (42 days), when this parameter reached a value less than or equal to 1 MPN/100 mL (see Figure 3a).

During peak contamination 1, the effluent from all SSFs showed an increase in the total coliform count of approximately one order of magnitude compared to the last days of the ripening period. Total coliforms in the raw water reached values of 1.18 × 10^5^ and 1.13 × 10^5^ MPN/100 mL in the Paranoá lake water and the lake water with dissolved CYN, respectively. These values represented an increase of two orders of magnitude concerning the raw water of the last days of ripening. Hendricks and Bellamy [49] indicate a direct correlation between the density of total coliforms in raw water and filtered water. Consequently, this may have been one of the causes of the rise in total coliform levels in the filtered water during the first contamination peak.

The second contamination peak was applied three weeks later. In this period, the total coliform density in the effluent from the SSFs decreased to 1 MPN/100 mL or lesser values. Such behavior may indicate a diversification and evenness of the biofilm microbial community (*schmutzdecke*) over time. In the effluents from filters SSF3 and SSF4 exposed to CYN, the total coliform counts decreased to values less than or equal to 1 MPN/100 mL. This indicates that the cyanotoxin concentration in the raw water had no adverse effect on the removal efficiency of the filters. Thus, proper ripening and longer operating times were possibly the main contributing factors to this behavior. These results corroborate previous studies of Unger and Collins [34] and Haig et al. [44], who observed that the longer the SSF operation time favors the higher removal efficiency of different microbiological water quality parameters.

During the whole filtration run, the SSFs received, on average, raw water with an *E. coli* density of 152.18 MPN/100 mL. For *E. coli*, from the beginning of the operation, both pairs of SSFs that submitted to peaks of CYN and those not submitted produced filtered water with levels of this bacterium below the detection limit (1 MNP/100 mL); the results were not presented in Table A1, Table A2 and Table A3. This fact is likely due to the proliferation of the microbiota remaining in the sand after the scraping and resanding process of the SSFs before the start of operation. Crittenden et al. [50] argued that, after sequential filtration runs, the developed microbiota could establish along the first 15 cm depth of the filter media. These remaining microbiota may have been responsible for the reduction in *E. coli* from the beginning of the operation of the slow sand filtration system [51]. The absence of *E. coli* during the CYN contamination peaks indicates that this cyanotoxin did not significantly impact the microorganisms and mechanisms involved in its removal. This efficiency was maintained over the filtration run, evidencing the robustness of the slow sand filtration system for *E. coli* removal. *E. coli* counts are presented in Figure 3b.

**Figure 4:**
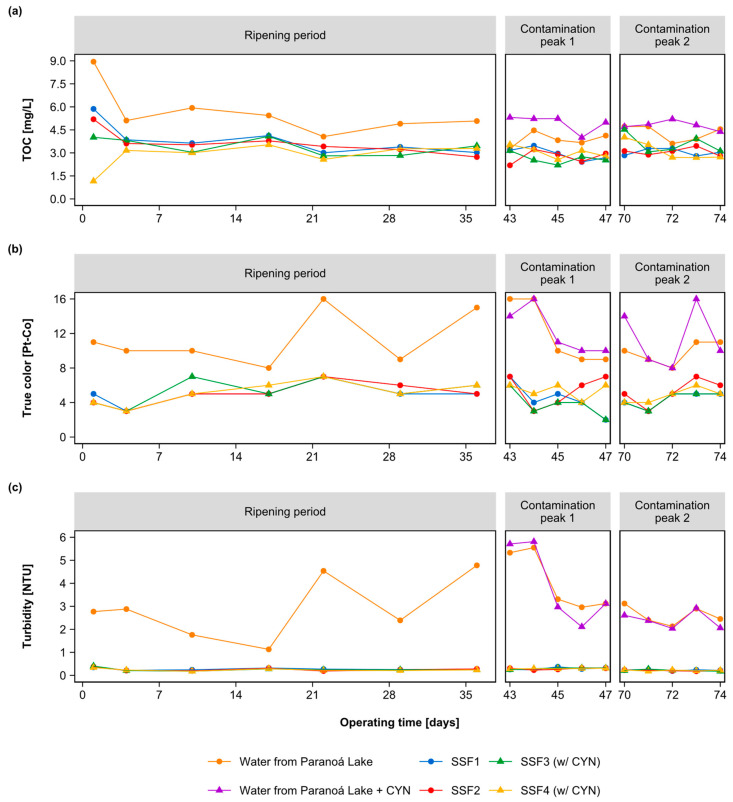
Water quality parameters monitored in the affluent and the effluent of each SSF during the ripening period and contamination peaks 1 and 2: (**a**) TOC concentration; (**b**) water true color; (**c**) turbidity.

Concerning water quality parameters, Figure 4 shows the values measured in the raw and filtered water in the three monitoring periods. The removal of TOC, true color, and turbidity remained consistent during the ripening period and contamination peaks, indicating CYN did not influence the efficiency of the filters SSF3 and SSF4. The average TOC concentration in the raw water ranged from 3.874 to 5.638 mg/L, and the average TOC removal by the SSFs was approximately 33%, resulting in an average residual TOC in the effluent of 3.165 mg/L. The average removal of true color was about 55%, with average values of 5 Pt-Co in the effluent from the SSFs. The SSFs’ effluent turbidities varied between 0.18 and 0.40 NTU, meeting the Brazilian standards for slow sand filtration (1 NTU) [52].

**Figure 5:**
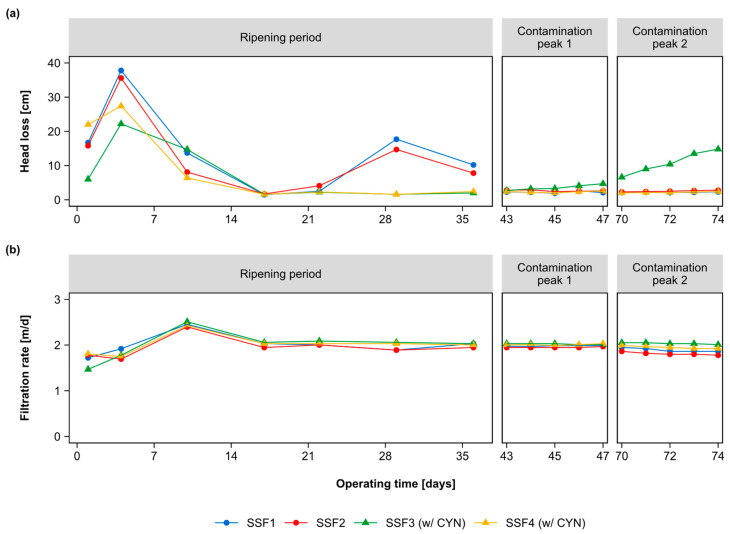
Hydraulic parameters monitored in each SSF during the ripening period and contamination peaks 1 and 2: (**a**) head loss in the first 5 cm from the top of the filter media; (**b**) filtration rate.

All the SSFs were variable in the ripening period regarding head loss. However, the head loss was stable in the contamination peaks and presented similar values among all SSFs (see Figure 5a). According to Haig et al. [44], the operating time of SSFs contributes to greater species diversity and evenness. This implies higher functional stability in SSFs. In this way, it is possible to explain the stabilization of the head loss over the filtration run of all SSFs. Only SSF3 showed an increase in head loss during the contamination peaks. Although the head loss of SSF3 was different from the other SSFs, in Figure 5b, it is observed that the filtration rate was not affected.

### 2.3. Comparative Analysis of the Performance of the Slow Sand Filters

During each monitoring period of the slow sand filtration system, the four SSFs were compared to identify possible differences. To assess differences over time, the performance of each SSF in each of the monitoring periods was also compared. Possible significant differences were checked using the Kruskal–Wallis non-parametric test. The tests with positive significance were submitted to Dunn’s multiple comparisons test to identify the different pairs.

**Figure 6:**
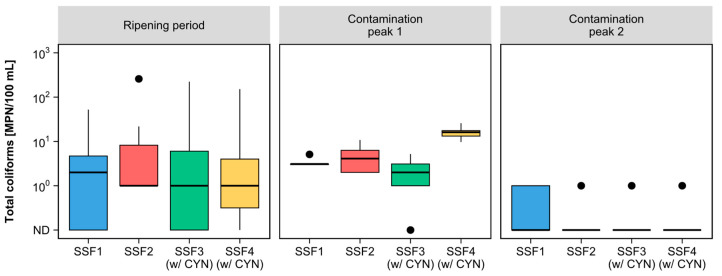
Box plots of the distribution of total coliform counts in the effluent of each SSF during the ripening period and contamination peaks 1 and 2. 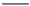 Median; 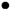 Outliers.

Concerning removing water quality parameters, the Kruskal–Wallis test did not reveal significant differences between the four SSFs over the filtration run. However, during contamination peak 1, there was a statistically significant difference in total coliform removal between the SSFs, with a *p*-value of 0.00961. Figure 6 compares the total coliform content distribution measured in the effluent of the SSFs during each period of the filtration run.

As shown in Figure 6, the distribution of the SSF4 data during the first contamination peak led to the differences indicated in the removal of total coliforms. However, the residuals from SSF3, also exposed to contamination peaks, were similar to the controls (SSF1 and SSF2). Therefore, regardless of the presence of CYN in the raw water, the filters achieved similar total coliform removal to the controls.

**Figure 7:**
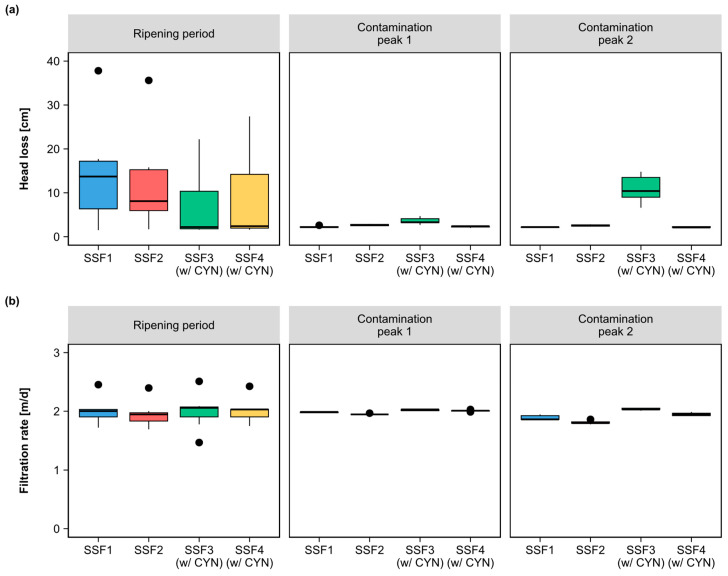
Box plots of the distribution of hydraulic parameter measurements monitored in each SSF during the ripening period and contamination peaks 1 and 2: (**a**) head loss in the first 5 cm from the top of the filter medium; (**b**) filtration rate. 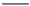 Median, 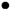 Outliers.

When analyzing the hydraulic parameters of each SSF, an apparent difference was exhibited in the head loss during the ripening period (Figure 7). However, the Kruskal–Wallis test showed that the observed variation was not statistically relevant.

Based on the results of Dunn’s test, presented in Table 2, the SSF3 was identified as the SSF with the most remarkable significant differences in head loss compared to the other SSFs evaluated. The differential increase in the head loss of SSF3, observed in the contamination peaks (Figure 7a), could be hypothetically attributed to the type of microorganisms established in the *schmutzdecke*, which occupied the empty spaces at the top of the filter media differently than the microorganisms that colonized the other SSFs. This occupation probably hindered the passage of water through the filter media and, as a result, increased head loss.

The differences observed between the SSFs in each period were mainly related to the hydraulic parameters. This indicates that regarding reaching the expected water quality parameters, the filters presented similar performances to each other despite the presence of CYN in the water.

**Figure 8:**
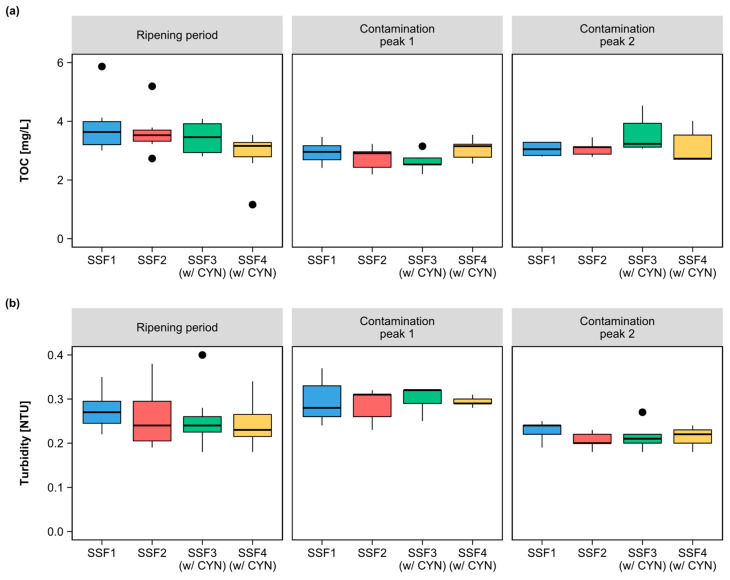
Box plots of the distribution of measurements of the water quality parameters monitored in the affluent and effluent of each slow sand filter during the ripening period and contamination peaks 1 and 2: (**a**) total organic carbon concentration; (**b**) turbidity. 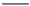 Median, 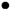 Outliers.

On the other hand, when comparing the performance of each SSF over the filtration run, significant differences were observed in the removal of TOC and turbidity (Figure 8 and Table 3). The concentration of CYN in the effluent did not significantly affect the removal of TOC. Although the TOC residuals in the SSF3 and SSF4 filters showed greater variability in contamination peak 2 (Figure 8a), the filters’ medians remained close in all periods, and no marked differences were observed between the two contamination peaks.

Turbidity removal showed significant differences between contamination peak 1 and contamination peak 2 in all SSFs. The difference was due to reduced turbidity levels at the second contamination peak. This behavior is expected in slow sand filtration systems. The deposition of particulate material over time at the top of the filter media decreases the empty spaces and increases the retention of the turbidity particles [53].

### 2.4. Evaluation of Cylindrospermopsin Removal

**Figure 9:**
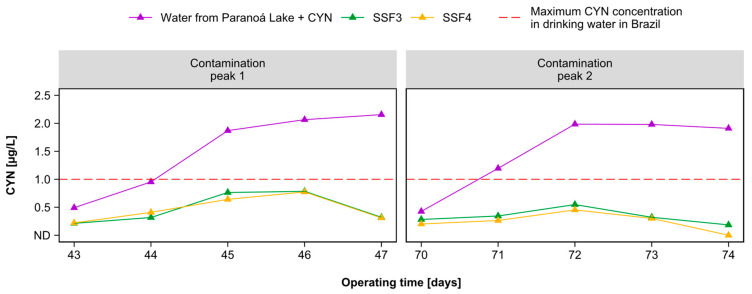
CYN concentration measured in the effluents of filters SSF3 and SSF4 during contamination peaks 1 and 2.

The results of CYN removal in filters SSF3 and SSF4 are shown in Figure 9. The results revealed the efficiency of filters SSF3 and SSF4 after ripening them. The ripening of both SSFs submitted to peaks of CYN ensured the stability of the biological activity at the filter media, which contributed to reducing the cyanotoxin level in the SSF effluents. This evidence suggests that the stable biological activity at the filter media played a crucial role in efficiently removing CYN.

Both SSFs removed more than 33% of CYN during contamination peaks from the first day of each peak application. Furthermore, the CYN removal gradually raised over time, even as the CYN level in the raw water increased. The removal was higher during the second contamination peak applied three weeks after the first peak, suggesting that the SSFs’ colonizing microbiota became more stable and efficient with the SSFs’ operation time, as already proven in other studies [44]. It is important to note that the operation time of SSFs played a crucial role in removal efficiency, allowing microbiota diversification, stabilizing biological activity, and adapting microorganisms to fluctuations in the quality of the inflowing water.

During contamination peak 1, the maximum CYN residual levels in the effluent from SSF3 and SSF4 were 0.784 µg/L and 0.774 µg/L (62.03% and 62.52% removal efficiency), respectively. In contamination peak 2, the maximum CYN residuals were 0.548 µg/L and 0.453 µg/L (72.39% and 77.18% removal efficiency), respectively. At the end of contamination peak 2, SSF4 removed CYN at a level not detectable. Notably, in both contamination peaks, the concentrations of CYN in the effluent from SSF3 and SSF4 remained below the limit of 1 µg/L established by Brazilian legislation for drinking water [52].

These results demonstrate that slow sand filtration technology can effectively remove CYN to acceptable levels established for drinking water in Brazil. This study achieved these results when the concentration of CYN in the raw water was approximately 2 µg/L or lower, and the SSFs were properly ripened. These results are also promising concerning international standards recommended by the WHO of 0.7 µg/L for the CYN concentration in drinking water [54].

Regarding the mechanisms contributing to the CYN removal, the adsorption evaluation on the sand used as filter media did not show interactions of the cyanotoxin with sand, suggesting that such a mechanism was not involved in CYN removal. On the other hand, biodegradation seems to be a strongly possible removal mechanism. However, in this study, the effluents from SSF3 and SSF4 were not submitted for investigation to identify potential by-products of CYN biodegradation. Besides biodegradation, this study did not prove other CYN removal mechanisms, such as adsorption on the biofilm [55].

### 2.5. Characterization of the Microbiota in Slow Sand Filters

**Figure 10:**
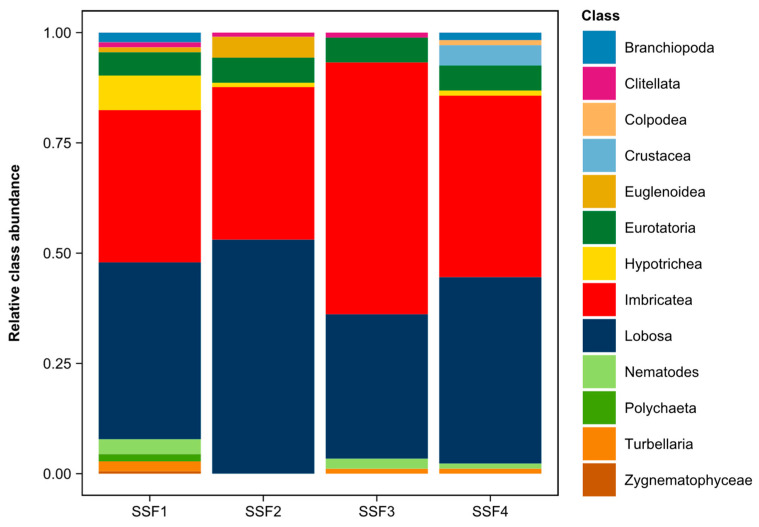
Relative abundance of the taxonomic classes identified in each SSF at the end of the filtration run.

Optical microscopy analysis of the microbiota colonizing the SSFs throughout the filtration run identified 13 taxonomic classes from different taxonomic kingdoms. Figure 10 shows the relative abundance of these classes in each SSF at the end of the filtration run.

Some of the classes identified on the SSFs, among others, have been previously recorded in the Paranoá Lake microbial community [56]. In addition, in previous studies, these same classes were also identified in the system of slow sand filtration using this lake water [45,47,57,58].

The relative abundance of the classes in the SSFs did not necessarily reflect their relative abundance in the Paranoá Lake. The composition of the microbiota occurring in the SSFs was influenced by the specific environmental and operational conditions predominant in the ecosystem of the SSFs [44,59]. Considering this, in all SSFs, protozoa of the Imbricatea class and amoebas of the Lobosa class were predominant. However, the proportion of these classes varied in the SSFs exposed and unexposed to CYN during contamination peaks. In the unexposed SSFs, the Lobosa class predominated over Imbricatea, while in the exposed SSFs, the Imbricatea class showed a higher proportion than the Lobosa class. SSF1 and SSF2 exhibited a proportion of 40.11% and 53.08% of the Lobosa class, respectively, while in SSF3 and SSF4, it was 32.77% and 42.29%, respectively. On the other hand, the proportion of the Imbricatea class in SSF1 and SSF2 was 34.54% and 34.60%, respectively, while in SSF3 and SSF4, it was 57.06% and 41.14%, respectively. The higher proportion of the Imbricatea class in the SSFs exposed to CYN suggests its lower sensitivity to this cyanotoxin when compared to the Lobosa class.

Among the representatives of the Imbricatea class, protozoa from the genus *Euglypha*, which occur naturally in aquatic environments with little contamination [38], were the most dominant. These organisms are more abundant in oxygen-rich aquatic environments with a high diversity of planktonic species [38]. Species of the genus *Euglypha* play an essential role in the ecosystem, feeding on bacteria, algae, and other microorganisms present in the environment in which they occur.

Lobosa class representatives in the SSFs included genera *Arcella*, *Centropyxis*, and *Amoeba* microorganisms. These species are found in various aquatic habitats, such as lakes, ponds, wetlands, and rivers. Species of the genus *Arcella* are often found in sediments or decomposing vegetation, where they feed on bacteria, algae, and organic debris. Species of the genus *Centropyxis* are commonly found in sediments and the surface layer of humid soils, feeding on suspended organic particles and bacteria. The genus *Amoeba* can occur in various aquatic environments and feeds mainly on bacteria, algae, and other microorganisms. Notably, most of the microbiota found in SSFs are native to the sediment. Thus, finding it on top of the filter media is natural as this superior section can be a relatively similar substrate in the SSFs.

The high removal efficiency of total coliforms and *E. coli* in the SSFs can be attributed to the occurrence and predominance of species belonging to the classes Imbricatea and Lobosa, which feed mainly on bacteria and suspended organic particles, thus suggesting a predation mechanism of removal. Interestingly, despite the slightly different proportions, the predominant classes were the same in all SSFs, indicating that dissolved CYN did not significantly affect the high removal efficiency of total coliforms and *E. coli*.

The Eurotatoria class, represented by the Digononta, Monogononta, and *Lecane* groups, was found in the SSFs in proportions between 5% and 6%. These microorganisms, known as rotifers, are widely distributed in freshwater aquatic habitats such as lakes, ponds, rivers, streams, and wetlands. Their occurrence is often considered an indicator of good water quality.

Microorganisms of the Eurotatoria class feed on organic particles suspended in the water, such as algae, bacteria, organic detritus, and other microorganisms. Some rotifers are filter feeders with specialized structures, such as the ciliated crown, to capture food particles suspended in the water. In addition to contributing to nutrient cycling in aquatic ecosystems, rotifers also play an essential role in the population control of other microorganisms. Rotifers have also been of great sanitary interest. Studies have demonstrated the predation of *Cryptosporidium* oocysts by different species of this group of microorganisms [39,40]. The occurrence of rotifers in SSFs shows the potential for pathogen control that slow sand filtration offers in water treatment.

The studied SSFs presented similar microbiota compositions. However, in some SSFs, other taxonomic classes occurred, differentiating them. Thus, Shannon diversity indices were calculated for each SSF. The SSF1 had a Shannon diversity index of 1.57, while SSF2, SSF3, and SSF4 had indices of 1.10, 1.04, and 1.31, respectively. These results indicated that exposure to CYN does not appear to be a determining factor in the diversity of the microbiota of the SSFs. SSF1 and SSF4, which were exposed and not exposed to CYN, respectively, had the highest Shannon diversity indices. This suggests that other factors may have played a more significant role in the observed diversity.

One of the main factors that possibly influenced the diversity of the microbiota was the bacterial community. In slow sand filters, bacteria contribute to the degradation of organic compounds and are a source of food for planktonic and benthonic organisms [36,60,61,62]. Alterations and differentiations in the bacterial community can influence the development of planktonic and benthic organisms due to trophic interactions between these groups. The lack of an analysis of the bacterial community did not allow the determination of its relationship with the composition of the remaining microbiota.

The positioning of the slow sand filtration system probably also influenced the differences in the microbiota diversity in the SSFs. The SSFs were arranged in increasing order of distance from one of the laboratory windows, with SSF1 being the closest and SSF4 the furthest. The SSF1 showed the highest diversity compared to the other SSFs. Despite all cares taken to prevent the incidence of light on the SSFs, the laboratory routine and the filtration system’s location allowed small plots of light to pass through, which likely promoted the occurrence of photosynthesizing microorganisms. The optical microscopy analysis of the microbiota from SSF1 revealed the occurrence of microalgae of the genus *Micrasterias* belonging to the class Zygnematophyceae. These microalgae represented approximately 0.56% of the taxonomic classes identified in this SSF (Figure 10). In addition to their contribution to oxygen production, microalgae of the genus *Micrasterias* are known to form colonies and biofilms [63], providing habitats, shelter, and food sources for a variety of aquatic organisms, such as small invertebrates and protozoa [64]. The presence of microalgae of the genus *Micrasterias* in the SSF1 may have been one of the factors contributing to the higher Shannon diversity index observed in this filter, also explaining the higher-class richness observed in SSF1 compared to the other SSFs.

**Figure 11:**
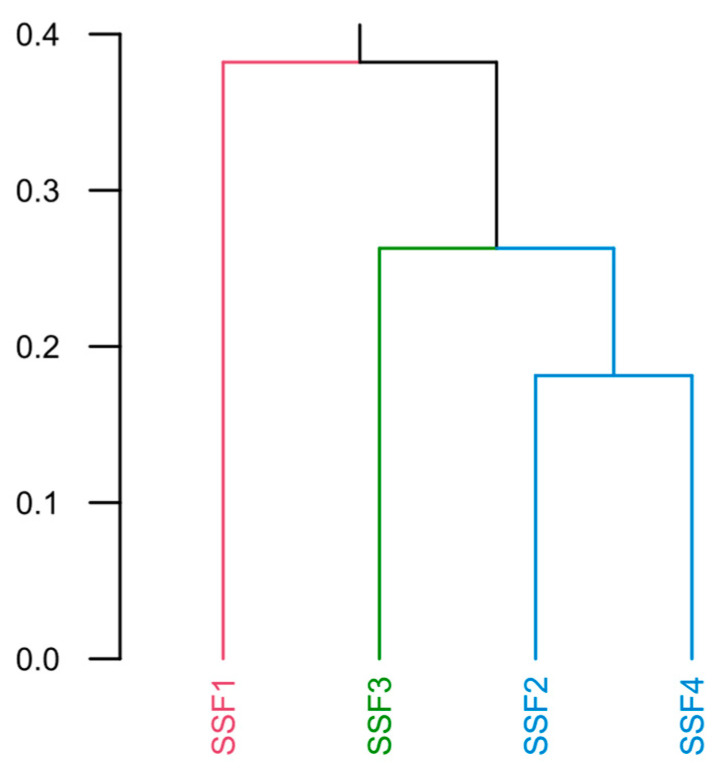
Cluster dendrogram of the results of the Bray–Curtis dissimilarity analysis of SSF diversity.

The results of the Bray–Curtis dissimilarity analysis showed that SSF1 and SSF3 were the most differentiated among the four filters evaluated. Figure 11 depicts the dendrogram of the data clustering, showing the dissimilarity in the community diversity in filters SSF1 and SSF3 compared to SSF2 and SSF4. The SSF1 stood out as the most dissimilar due probably to a combination of several factors, including the filtration system’s location, the incidence of light that favored photosynthesizing microorganisms developing, the formation of colonies and biofilms by microalgae of the genus *Micrasterias*, and the consequent creation of habitats and shelter for other aquatic organisms. In contrast, SSF3 showed the lowest class richness among the SSFs evaluated, which may have contributed to its distinction from the others.

## 3. Final Considerations

For the first time, this study investigated the removal of CYN by ripened SSFs treating surface water and the toxin effects on removal efficiency of physical, chemical, and biological water quality parameters.

The inhibition of light incidence caused an increase, by two times, in the time required to reach ripening in SSFs supplied with water from Paranoá Lake. The light limitation resulted in a restriction of phytoplankton primary metabolism, impairing the development of aerobic microorganisms key to ripening.

During the first contamination peak, total coliform counts increased in all filter effluents. The increase in the density of total coliforms in the raw water was the possible leading cause, rather than the presence of CYN. As the *schmutzdecke* developed, an improvement in removing this group of bacteria was observed.

The ripening period of SSFs plays an essential role in improving water quality parameters. CYN did not interfere with removing *E. coli*, TOC, true color, and turbidity in ripened SSFs. Still, in the context of its performance, the concentration of CYN in the effluent of the SSFs met the requirements of 1 µg/L established by Brazilian legislation for drinking water. The degradation of CYN is influenced by abiotic factors such as sunlight, which is effective in the presence of pigments and algogenic material [65]. On the other hand, water pH and temperature do not promote effective cyanotoxin degradation [65]. However, in biological processes, pH and temperature are determinants of degradation efficiency. Temperatures between 20 and 30 °C and pH between 6.5 and 8.0 result in the highest biodegradation capacity [66,67,68,69,70]. Considering this, under the experimental conditions used in this study, the raw water quality and the CYN removal observed during the contamination peaks suggest that biodegradation was probably one of the most influential mechanisms in CYN removal. In addition, the filter media acted as a support medium, which, according to Klitzke et al. [71], allows the proliferation of microorganisms with the potential to degrade the cyanotoxin. However, other mechanisms, like adsorption on biofilm, not evaluated herein, may have contributed to removing the toxin. In this regard, it is essential to perform further studies to elucidate the mechanisms that act in removing CYN.

The analysis of the microbiota at the end of the filtration run revealed the predominance of protozoa of the genus *Euglypha*, belonging to the class Imbricatea, and amoebae of the genera *Arcella*, *Centropyxis*, and *Amoeba*, belonging to the class Lobosa. These microorganisms were crucial in removing total coliforms and *E. coli* through their trophic interactions. Although in smaller proportions, the presence of rotifers in the SSFs was also of great importance, as it shows the potential of this water treatment technology controlling pathogens in water, such as *Cryptosporidium*. The exposure to CYN did not have a significant impact on microbiota composition. However, some differences were observed between the SSFs, mainly attributed to the presence of microalgae of the genus *Micrasterias*. Through photosynthesis and biofilm formation, these microalgae probably provided favorable conditions for developing a more diverse community in the *schmutzdecke*.

## 4. Materials and Methods

### 4.1. Cylindrospermopsin Standard

This study used a CYN solid standard to carry out the filtration experiments. A 500 µg solid standard of CYN, with purity > 95%, was purchased from Eurofins/Abraxis (Eurofins/Abraxis, Warminster, PA, USA). From this standard, solutions of the cyanotoxin were prepared and inoculated into the raw water to simulate contamination peaks.

### 4.2. Raw Water

Two raw waters were prepared to evaluate the SSF efficiency in removing CYN. Water from Paranoá Lake, an artificial lake in Brasília/DF, Brazil, was the matrix chosen to prepare both raw waters.

Recent studies did not identify CYN-producing cyanobacteria in Paranoá Lake [72]. Furthermore, in the study by Abbt-Braun et al. [73], this cyanotoxin was not detected in samples of lake water analyzed by LC-MS/MS. In this study, the LC-MS/MS method analyses did not identify the presence of CYN in the raw water.

The contaminated raw lake water received the CYN standard solutions. Non-contaminated raw lake water served as a control. Contaminated raw water was used during the contamination peaks at concentrations between 0.5 and 2 µg/L. Section 4.4 describes how these peaks were applied.

### 4.3. Pilot Scale System of Slow Sand Filtration

**Figure 12:**
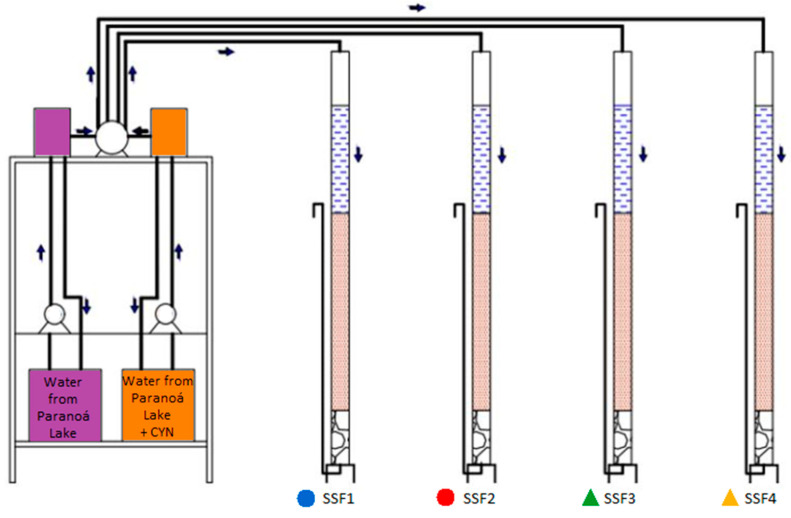
Pilot-scale slow sand filtration system used in this work.

The slow sand filtration system was composed of the following: (1) two 60 L reservoirs for feeding the raw water, one for the Paranoá Lake water and the other one for the Paranoá Lake water inoculated with CYN solution; (2) two intermediate reservoirs of constant level and with a tulip shape spillway; (3) two dosing pumps; (4) a four-channel peristaltic pump; and (5) four SSFs, constructed in acrylic columns (SSF1, SSF2, SSF3, SSF4). Figure 12 shows the pilot-scale slow sand filtration system used in this work.

The head loss in the SSFs was measured using eleven piezometric tubes distributed along the filter media. To reduce the incidence of light and to avoid photodegradation of CYN [65], aluminum foils covered the external part of the columns during system operation. The filter media used in this study was sand with an effective size of 0.25 mm, grain size ranging from 0.15 to 1 mm, coefficient of uniformity of 2.38, porosity of 0.4, and a filter bed depth of 105 cm. The support layer had a thickness of 30 cm and was composed of three sub-layers, each 10 cm thick. The gravel used in the support layer had a size ranging from 1 to 31.7 mm. The top sub-layer had a size range of 1–2 mm, the intermediate sub-layer had a size range of 4.76–6.30 mm, and the bottom sub-layer had a 19.1–31.7 mm size range.

This study used the slow sand filtration system operated by Sá et al. [74] in previous years with some modifications. A few weeks before this experiment, another study used the filtration system. Therefore, the filter media was prepared before starting. All the SSFs were subjected to a scraping and resanding process to ensure proper preparation of the filter media. The scraping consisted of removing the first 3 cm of the top of the filter media from each SSF. It is significant to mention that the previous use of the slow sand filtration system may have left microorganism inoculum on the filter media, which may have grown and developed throughout the experiment conducted in the present study. For reference purposes, SSF1 and SSF2 were fed only with water from Paranoá Lake, both in the previous experiments and in the experiments performed in this study. In the same way, SSF3 and SSF4 received water from Paranoá Lake containing dissolved CYN during the last and in this study’s experiments.

### 4.4. Operation of Slow Sand Filters

SSFs operated with a filtration rate of approximately 2 m/d, controlled by a constant filtration rate with variable water levels. The experimental hydraulic retention time of 14 h was determined by tracer tests, as described in the following section. The system operated for 74 days, with daily renewal of the raw water. During the first 42 days of operation, considered the ripening period, all filters received water from Paranoá Lake free of CYN. From day 43 to day 47, the first CYN contamination peak occurred, which lasted for a total of 5 days. Two of the four filters received the contaminated water (SSF3 and SSF4), while the other two continued receiving water from Paranoá Lake without CYN (SSF1 and SSF2).

During the period between CYN contamination peaks, from day 48 to day 69, the influent of all SSFs was water from Paranoá Lake without CYN. The second CYN contamination peak occurred from day 70 to day 74, lasting 5 days. Again, SSF3 and SSF4 received the CYN-contaminated water, while SSF1 and SSF2 continued to receive water from Paranoá Lake without toxin.

The contamination peaks started with a concentration of CYN in the raw water around 0.5 µg/L. Thereafter, the concentration was increased stepwise during the first three days of each contamination peak, representing the exponential growth of cyanotoxin-producing cyanobacteria. After this increase, the concentration of CYN remained constant for another two days, indicating the stationary phase of the bloom. During this phase, the concentration of CYN in the raw water reached approximately 2 µg/L, a value frequently found in Brazilian aquatic environments.

The experiments were conducted using contamination peaks to simulate natural aquatic environments in which the concentration of dissolved CYN is not constant over time. This approach was used to simulate the behavior of CYN production during the natural growth cycle of CYN-producing cyanobacteria, as studied by Davis et al. [75], which showed the relationship between growth phases and the concentration of dissolved CYN.

### 4.5. Hydraulic and Tracer Test

A hydraulic evaluation was conducted simultaneously with the four SSFs during the tracer test. The total head loss of each filter was measured every 60 min, along with conductivity. The data collected for head loss were then subjected to a Kruskal–Wallis test to determine if there were any statistically significant hydraulic differences. The tracer test involved diluting NaCl in distilled water until the solution reached a conductivity of 850 µS/cm. The conductivity was measured in the NaCl solution and the effluent of each filter. The test concluded once the effluent conductivity reached 850 µS/cm.

### 4.6. Tests of Cylindrospermopsin Adsorption on Sand Filter Media

The sand’s capacity to adsorb CYN was evaluated by applying OECD Test 106 [76]. The assays consisted of stirring samples of sand suspension in an SI600R orbital shaker (Lab Companion) with a constant temperature of 22 °C. Before the adsorption assays, a suspension was prepared by adding a 10 g fraction of sand to 45 mL of a CaCl_2_ solution and stirring for at least 12 h to stabilize before the adsorption test. After this period, the suspension received 5 mL of CYN concentrated solution. The final concentrations of CaCl_2_ and CYN in the 50 mL solution were approximately 0.01 M and 30 µg/L, respectively. Four samples were analyzed: a control consisting of a CaCl_2_ solution with CYN and without sand addition; a blank consisting of the 50 mL CaCl_2_ solution 0.01 M and 10 g of sand without CYN addition; and two samples of 10 g of sand suspended in the CaCl_2_ solution with the addition of CYN. The control was used to verify the stability of CYN in the CaCl_2_ solution and as a reference for the samples containing sand. The blank was used to detect compounds in the sand that may interfere with CYN analysis. The test was performed in duplicate to corroborate the results obtained.

### 4.7. Sample Collection, Storage, and Analysis

In this study, ten parameters were monitored, including water quality parameters and operational parameters. The monitored parameters were (1) head loss, (2) filtration rate, (3) total coliforms, (4) *E. coli*, (5) alkalinity, (6) true color, (7) pH, (8) turbidity, (9) TOC, and (10) CYN. During the SSF ripening, all parameters were monitored weekly. During the contamination peaks, all parameters were monitored daily. The filtered water samples were collected 15 h after the raw water analyses. All water samples were analyzed immediately after collection and not stored. Only the samples for detection and quantification of CYN were stored for possible confirmation of results. These samples were kept in pre-sanitized 10 mL glass vials at −20 °C for 30 days. The head loss was measured using piezometric tubes distributed along the filter media. The filtration rate was measured indirectly by the ratio between the effluent flow rate and the cross-sectional area of each filter. Total coliforms and *E. coli* were quantified by the Colilert test from IDEXX. Alkalinity was measured in the raw water only, using neutralization volumetry following procedure 2320 of the “Standard Methods for the Examination of Water and Wastewater”. True color was measured by spectrophotometry using a HACH DR 5000 spectrophotometer. Turbidity measurements were performed by using a HACH 2100 NA turbidimeter. TOC was measured on a Shimadzu 500 carbon analyzer. CYN was measured by LC-MS/MS using the same equipment and following the same procedure described by Ferreira et al. [27].

### 4.8. Analysis of the Planktonic and Benthonic Community

Microbiological analyses focused on the planktonic and benthonic communities. Although not analyzed, the bacterial community was probably primarily responsible for removing CYN and TOC. However, the analysis performed herein allowed for identifying the effects of CYN on the protists and metazoa community, given its role in removing coliform bacteria, *E. coli*, and pathogens such as *Cryptosporidium* oocysts.

The sand samples were collected after 74 days of operation of the filters. For the sampling, the water over each filter’s media was drained by siphoning, leaving only a minimal water layer to keep the filter media submerged. The sand from the first 3 cm from the top of the filter media was collected while still wet and transferred to 1 L Erlenmeyer flasks previously sterilized in an autoclave.

Each Erlenmeyer flask contained 500 mL of Paranoá Lake water previously filtered through a 0.22 µm cellulose ester membrane. After filtering, the water was sterilized in an autoclave. This water was used to minimize the stress of the microfauna present in the collected sand. The Erlenmeyer flasks containing the water and sand were shaken on a SI600R orbital shaker (Lab Companion) for 2 min at 150 rpm. Then, the Erlenmeyer flasks were left to stand for 30 s to allow part of the sand to sediment. After the resting period, aliquots of the supernatant were taken from the flasks.

The aliquots were then placed in a Sedgewick–Rafter chamber to visualize the microorganisms by light microscopy using a Leica optical microscope model DM LB2. When necessary, the samples were diluted in the same filtered and sterilized Paranoá Lake water. Finally, the microorganisms identified in the samples of each filter were counted and classified using Streble and Krauter [38] as a reference.

## Figures and Tables

**Table 1:** Results of the two-way ANOVA to verify significant differences in the results of the adsorption evaluation of CYN on sand used as a filter media.

Effect	% of Total Variation	Mean Squares	F Value	*p*-Value
Sample (control, samples 1 and 2)	1.62	0.7905	0.146	0.865
Replica	20.96	20.4800	3.791	0.072

**Table 2:** Filter pairs that were different in Dunn’s multiple comparisons test, separated by parameter, within each period of the slow sand filtration system operation.

Period	Parameter	Group 1	Group 2	Z-Value	Adjusted *p*-Value
Contamination peak 1	Head loss 5 cm	SSF1	SSF3	3.21	0.00788
		SSF3	SSF4	−2.97	0.0177
	Filtration rate	SSF2	SSF3	3.75	0.00108
		SSF2	SSF4	2.90	0.0220
Contamination peak 2	Head loss 5 cm	SSF1	SSF3	3.27	0.00647
		SSF3	SSF4	−3.32	0.00535
	Filtration rate	SSF2	SSF3	3.96	0.000458

**Table 3:** Water quality parameters with significant differences in Dunn’s multiple comparisons tests between the contamination peaks in each filter.

Filter	Parameter	Group 1	Group 2	Z-Value	Adjusted *p*-Value
SSF1	Turbidity	Peak 1	Peak 2	−2.37	0.0538
SSF2	Turbidity	Peak 1	Peak 2	−2.46	0.0422
SSF3	TOC	Peak 1	Peak 2	2.44	0.0438
	Turbidity	Peak 1	Peak 2	−2.65	0.0244
SSF4	Turbidity	Peak 1	Peak 2	−2.58	0.0296

## Data Availability

The data presented in this study are available on request from the corresponding author.

## Appendix A

**Table A1:** Water quality parameters measured in raw water and filtered water during the ripening period.

Parameter	Raw Water (w/o. CYN)	SSF1	SSF2	SSF3	SSF4
**CYN (µg/L)**					
Min.	ND	ND	ND	ND	ND
Max.	ND	ND	ND	ND	ND
Mean	ND	ND	ND	ND	ND
SD	ND	ND	ND	ND	ND
% removal	-	-	-	-	-
**Head loss 5 cm (cm)**					
Min.	-	1.5	1.7	1.6	1.6
Max.	-	37.8	35.6	22.2	27.4
Mean	-	14.3	12.5	7.2	9.1
SD	-	12.2	11.4	8.1	10.9
**TOC (mg/L)**					
Min.	4.061	3.009	2.733	2.808	1.161
Max.	8.945	5.866	5.193	4.085	3.531
Mean	5.638	3.843	3.643	3.437	2.855
SD	1.565	0.983	0.763	0.551	0.804
% removal	-	31.30	34.04	36.99	44.96
**Total coliforms (MPN/100 mL)**					
Min.	488.4	0.0	1.0	0.0	0.0
Max.	14,428.8	52.4	258.2	223.8	151.8
Mean	7556.1	9.6	41.0	34.3	24.4
SD	5308.1	19.3	96.1	83.7	56.5
Log removal	-	3.20	3.10	2.96	3.01
**True color (Pt-Co)**					
Min.	8	3	3	3	3
Max.	16	7	7	7	7
Mean	11	5	5	5	5
SD	3	1	1	1	1
% removal	-	54.20	53.91	51.69	52.76
**Turbidity (NTU)**					
Min.	1.13	0.22	0.19	0.18	0.18
Max.	4.78	0.35	0.38	0.40	0.34
Mean	2.89	0.27	0.26	0.26	0.24
SD	1.35	0.05	0.07	0.07	0.05
% removal	-	87.96	88.59	88.99	89.31

ND—Not detected.

**Table A2:** Water quality parameters measured in raw water and filtered water during the contamination peak 1.

Parameter	Raw Water (w/o. CYN)	SSF1	SSF2	Raw Water (w/ CYN)	SSF3	SSF4
**CYN (µg/L)**	ND	ND	ND			
Min.	ND	ND	ND	0.492	0.213	0.220
Max.	ND	ND	ND	2.155	0.784	0.774
Mean	ND	ND	ND	1.507	0.480	0.471
SD	ND	ND	ND	0.742	0.272	0.231
% removal	-	-	-	-	65.95	65.23
**Head loss 5 cm (cm)**						
Min.	-	1.9	2.4	-	2.7	2.0
Max.	-	2.6	2.9	-	4.7	2.5
Mean	-	2.2	2.6	-	3.6	2.3
SD	-	0.3	0.2	-	0.8	0.2
**TOC (mg/L)**						
Min.	3.276	2.413	2.191	4.001	2.204	2.560
Max.	4.469	3.469	3.229	5.312	3.150	3.539
Mean	3.874	2.940	2.745	4.952	2.631	3.049
SD	0.453	0.410	0.422	0.545	0.349	0.385
% removal	-	23.48	29.36	-	46.17	37.69
**Total coliforms (MPN/100 mL)**						
Min.	5410.8	3.0	2.0	5961.9	0.0	9.7
Max.	118,185.9	5.1	10.8	112,574.7	5.2	25.9
Mean	39,849.5	3.5	5.0	38,296.4	2.3	16.5
SD	47,130.2	0.9	3.7	44,811.7	2.0	6.1
Log removal	-	3.81	3.73	-	4.09	3.13
**True color (Pt-Co)**						
Min.	9	2	3	10	2	4
Max.	16	7	7	16	6	6
Mean	12	4	5	12	4	5
SD	4	2	2	3	1	1
% removal	-	62.92	50.61	-	68.41	54.27
**Turbidity (NTU)**						
Min.	2.96	0.24	0.23	2.11	0.25	0.28
Max.	5.55	0.37	0.32	5.81	0.32	0.31
Mean	4.05	0.30	0.29	3.94	0.30	0.29
SD	1.27	0.05	0.04	1.70	0.03	0.01
% removal	-	91.92	92.29	-	90.89	91.24

ND—Not detected.

**Table A3:** Water quality parameters measured in raw water and filtered water during the contamination peak 2.

Parameter	Raw Water (w/o. CYN)	SSF1	SSF2	Raw Water (w/ CYN)	SSF3	SSF4
**CYN (µg/L)**	ND	ND	ND			
Min.	ND	ND	ND	0.426	0.185	ND
Max.	ND	ND	ND	1.985	0.548	0.453
Mean	ND	ND	ND	1.499	0.336	0.244
SD	ND	ND	ND	0.686	0.133	0.165
% removal	-	-	-	-	70.30	78.54
**Head loss 5 cm (cm)**						
Min.	-	2.1	2.3	-	6.6	2.0
Max.	-	2.3	2.8	-	14.8	2.4
Mean	-	2.2	2.5	-	10.9	2.2
SD	-	0.1	0.2	-	3.3	0.2
**TOC (mg/L)**						
Min.	3.605	2.799	2.793	4.384	3.068	2.694
Max.	4.723	3.288	3.453	5.205	4.532	4.018
Mean	4.299	3.051	3.074	4.791	3.577	3.135
SD	0.514	0.234	0.257	0.295	0.637	0.609
% removal	-	28.06	27.27	-	25.09	34.32
**Total coliforms (MPN/100 mL)**						
Min.	6713.4	0.0	0.0	3156.3	0.0	0.0
Max.	12,174.3	1.0	1.0	12,174.3	1.0	1.0
Mean	8897.8	0.4	0.2	6442.9	0.2	0.2
SD	2315.9	0.5	0.4	3480.9	0.4	0.4
Log removal	-	3.96	3.83	-	4.09	3.63
**True color (Pt-Co)**						
Min.	8	3	3	8	3	4
Max.	11	5	7	16	5	6
Mean	10	4	5	11	4	5
SD	1	1	1	3	1	1
% removal	-	54.65	47.20	-	58.87	55.40
**Turbidity (NTU)**						
Min.	2.13	0.19	0.18	2.04	0.18	0.18
Max.	3.12	0.25	0.23	2.92	0.27	0.24
Mean	2.60	0.23	0.21	2.40	0.22	0.21
SD	0.40	0.02	0.02	0.37	0.03	0.02
% removal	-	91.14	91.94	-	90.85	90.89

ND—Not detected.

**Table A4:** Taxonomic classification of the microorganisms identified in each filter at the end of the filtration run.

Filter	Kingdom	Phylum	Class	Order	Family	Genus
SSF1	Animalia	Annelida	Clitellata	Oligochaeta		
SSF1	Animalia	Annelida	Polychaeta		Aeolosomatidae	*Aeolosoma*
SSF1	Animalia	Arthropoda	Branchiopoda			
SSF1	Animalia	Nematoda	Nematodes			
SSF1	Animalia	Platyhelminthes	Turbellaria			
SSF1	Animalia	Rotifera	Eurotatoria	Bdelloideia		
SSF1	Animalia	Rotifera	Eurotatoria	Monogononta		
SSF1	Animalia	Rotifera	Eurotatoria	Ploima	Lecanidae	*Lecane*
SSF1	Chromista	Ciliophora	Hypotrichea	Euplotida	Aspidiscidae	*Aspidisca*
SSF1	Chromista	Ciliophora	Hypotrichea	Euplotida	Euplotidae	*Euplotes*
SSF1	Plantae	Charophyta	Zygnematophyceae	Zygnematales	Desmidiaceae	*Micrasterias*
SSF1	Protozoa	Amoebozoa	Lobosa	Arcellinida	Arcellidae	*Arcella*
SSF1	Protozoa	Amoebozoa	Lobosa	Arcellinida	Centropyxidae	*Centropyxis*
SSF1	Protozoa	Amoebozoa	Lobosa			
SSF1	Protozoa	Euglenozoa	Euglenoidea	Euglenida	Euglenaceae	*Euglena*
SSF1	Protozoa		Imbricatea	Aconchulinida	Euglyphidae	*Euglypha*
SSF2	Animalia	Annelida	Clitellata	Oligochaeta		
SSF2	Animalia	Rotifera	Eurotatoria	Bdelloideia		
SSF2	Animalia	Rotifera	Eurotatoria	Ploima	Lecanidae	*Lecane*
SSF2	Chromista	Ciliophora	Hypotrichea	Euplotida	Euplotidae	*Euplotes*
SSF2	Protozoa	Amoebozoa	Lobosa	Amoebida	Amoebidae	*Amoeba*
SSF2	Protozoa	Amoebozoa	Lobosa	Arcellinida	Arcellidae	*Arcella*
SSF2	Protozoa	Amoebozoa	Lobosa	Arcellinida	Centropyxidae	*Centropyxis*
SSF2	Protozoa	Amoebozoa	Lobosa			
SSF2	Protozoa	Euglenozoa	Euglenoidea	Euglenida	Euglenaceae	*Euglena*
SSF2	Protozoa		Imbricatea	Aconchulinida	Euglyphidae	*Euglypha*
SSF3	Animalia	Annelida	Clitellata	Oligochaeta		
SSF3	Animalia	Nematoda	Nematodes			
SSF3	Animalia	Platyhelminthes	Turbellaria			
SSF3	Animalia	Rotifera	Eurotatoria	Monogononta		
SSF3	Animalia	Rotifera	Eurotatoria	Ploima	Lecanidae	*Lecane*
SSF3	Protozoa	Amoebozoa	Lobosa	Arcellinida	Arcellidae	*Arcella*
SSF3	Protozoa	Amoebozoa	Lobosa	Arcellinida	Centropyxidae	*Centropyxis*
SSF3	Protozoa	Amoebozoa	Lobosa			
SSF3	Protozoa		Imbricatea	Aconchulinida	Euglyphidae	*Euglypha*
SSF4	Animalia	Arthropoda	Branchiopoda			
SSF4	Animalia	Arthropoda	Crustacea			
SSF4	Animalia	Nematoda	Nematodes			
SSF4	Animalia	Platyhelminthes	Turbellaria			
SSF4	Animalia	Rotifera	Eurotatoria	Bdelloideia		
SSF4	Animalia	Rotifera	Eurotatoria	Monogononta		
SSF4	Animalia	Rotifera	Eurotatoria	Ploima	Lecanidae	*Lecane*
SSF4	Chromista	Ciliophora	Colpodea	Colpodida	Colpodidae	*Colpoda*
SSF4	Chromista	Ciliophora	Hypotrichea	Euplotida	Euplotidae	*Euplotes*
SSF4	Protozoa	Amoebozoa	Lobosa	Arcellinida	Arcellidae	*Arcella*
SSF4	Protozoa	Amoebozoa	Lobosa	Arcellinida	Centropyxidae	*Centropyxis*
SSF4	Protozoa	Amoebozoa	Lobosa			
SSF4	Protozoa		Imbricatea	Aconchulinida	Euglyphidae	*Euglypha*
